# Coronary Vasospasm Presenting as ST-Elevation Myocardial Infarction

**DOI:** 10.7759/cureus.22205

**Published:** 2022-02-14

**Authors:** Arminder Singh, Lam Nguyen, Stephanie Everest, Manoj Bhandari

**Affiliations:** 1 Internal Medicine, Cape Fear Valley Medical Center, Fayetteville, USA; 2 School of Medicine, Campbell University School of Osteopathic Medicine, Lillington, USA; 3 Cardiology, Cape Fear Valley Medical Center, Fayetteville, USA

**Keywords:** stress, ecg, st-elevation myocardial infarction (stemi), coronary artery spasm, vasospastic angina

## Abstract

Vasospastic angina (VSA), also known as Prinzmetal angina, is caused by reversible diffuse or focal vasoconstriction of coronary arteries. It is the most common diagnosis among patients presenting with signs of ischemia but no obstructive coronary artery disease. Patients with VSA can present with typical acute coronary syndrome (ACS) symptoms of chest pain or pressure, dyspnea, diaphoresis, fatigue, and syncope. VSA is a challenging diagnosis for physicians due to its nearly identical clinical presentation to active acute coronary syndrome patients. This report describes a similar diagnosis dilemma when a 69-year-old female presented with ST-elevation myocardial infarction (STEMI), who was eventually diagnosed with and treated for vasospastic angina.

## Introduction

Chest pain at rest is one of the most commonly presenting symptoms in the emergency room. For patients with abnormal acute electrocardiographic (ECG) changes, acute coronary syndrome (ACS) is highly suspected [1-2]. However, according to a 2008 study, 30% of these patients have unobstructed coronary arteries or an inappreciable lesion that could explain patients’ symptoms [3]. Possible etiologies for the non-obstructive coronary chest pain include vasospastic angina (VSA), takotsubo cardiomyopathy, coronary embolism, and myocarditis; and vasospastic angina is the most common diagnosis among them all. VSA is caused by a coronary artery spasm that is thought to be triggered by many different precipitating factors. Patients with VSA can present with typical ACS symptoms of chest pain or pressure, dyspnea, diaphoresis, fatigue, and syncope [1,3-5]. About 48% of VSA patients have acute ECG changes, and elevated cardiac markers were found in the minority of these patients. Risk factors of vasospastic angina include smoking, mental stress, alcohol consumption, substance abuse, and medication adverse effects such as beta-blockers, ergotamine, or pilocarpine [4-5]. Once the diagnosis of Prinzmetal’s angina is made, patients are commonly managed by medical therapy with great success. This case report presents the clinical manifestation, diagnosis, medical management, and outcomes of a vasospastic angina patient hospitalized with suspicion for myocardial infarction.

## Case presentation

**Figure 1:**
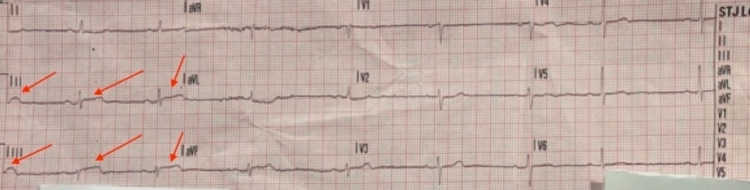
Electrocardiogram ( ECG) demonstrating ST-elevation inferior leads (II/III/aVF) and in aVL ST-elevation demonstrated by red arrows

A 69-year-old female with a history of rheumatoid arthritis presented to the emergency department (ED) via emergency medical services (EMS) for chest pain, heaviness, syncope, and diaphoresis. En route to the hospital, the ECG done by EMS showed inferior leads ST-segment elevation and ST-segment depression in lead I and aVL (Figure 1). Aspirin was given on the way to the hospital. On arrival to the ED, the patient was hemodynamically stable with an elevated blood pressure of 165/104 mmHg and heart rate of 60 beats per minute, and her symptoms had resolved. The patient’s labs showed elevated troponin I with a maximum value of 0.729 ng/ml. Due to the acute ECG changes and elevated troponin levels, the patient was admitted and evaluated for an ST-elevation myocardial infarction (STEMI).

**Figure 2:**
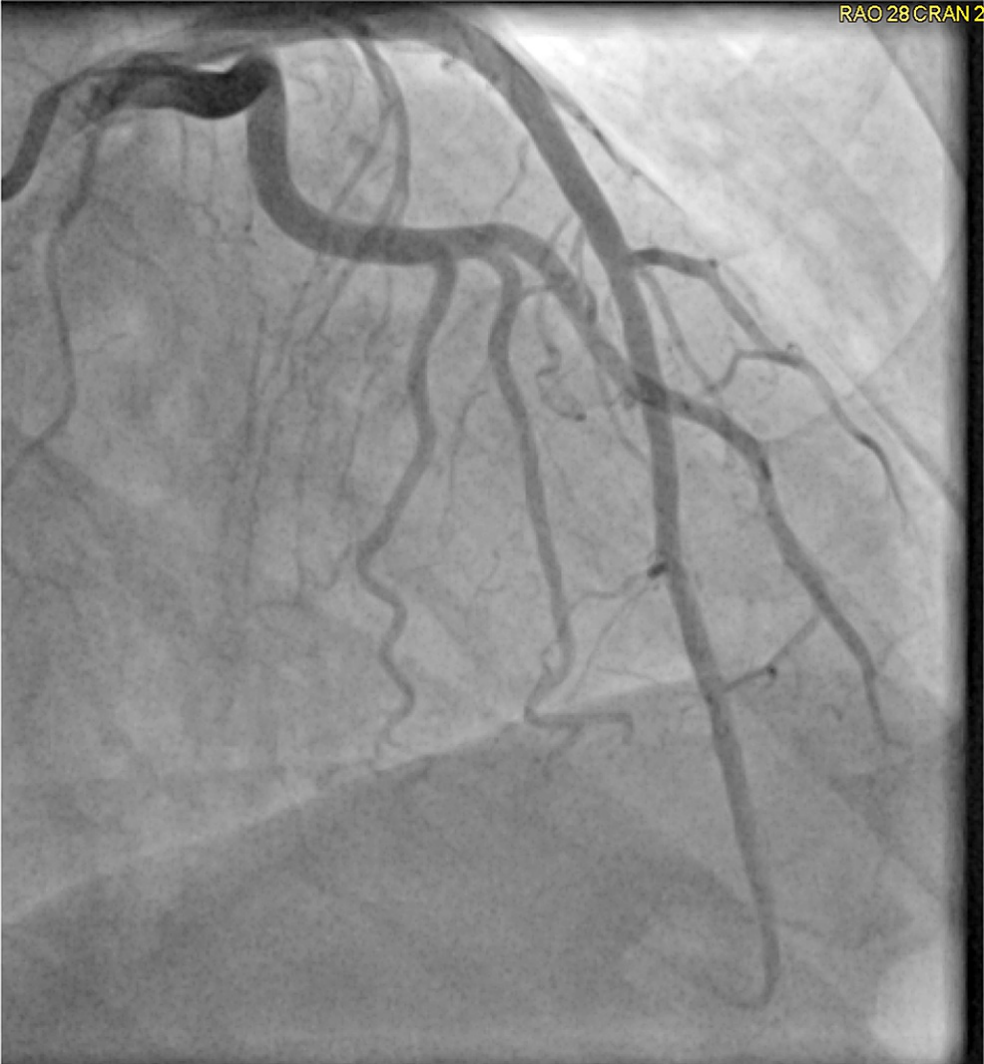
Coronary angiogram demonstrating no significant obstruction in left coronary artery circulation

**Figure 3:**
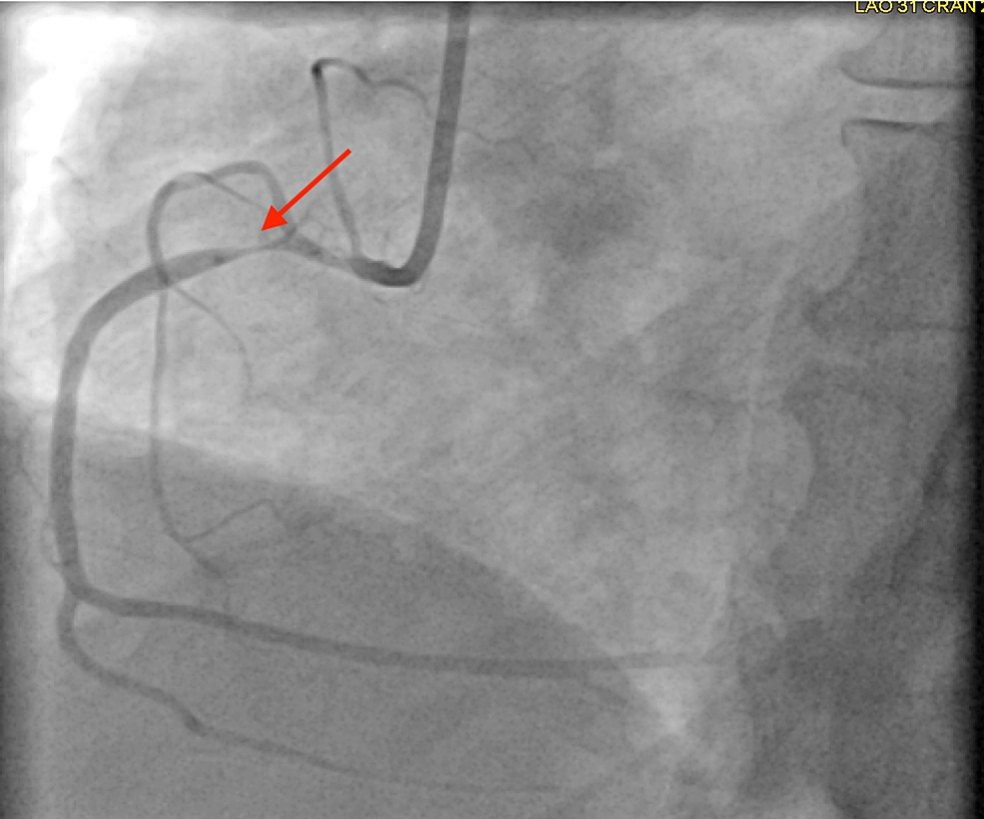
Coronary angiogram demonstrating right coronary artery obstruction Obstruction is demonstrated by the red arrow.

**Figure 4:**
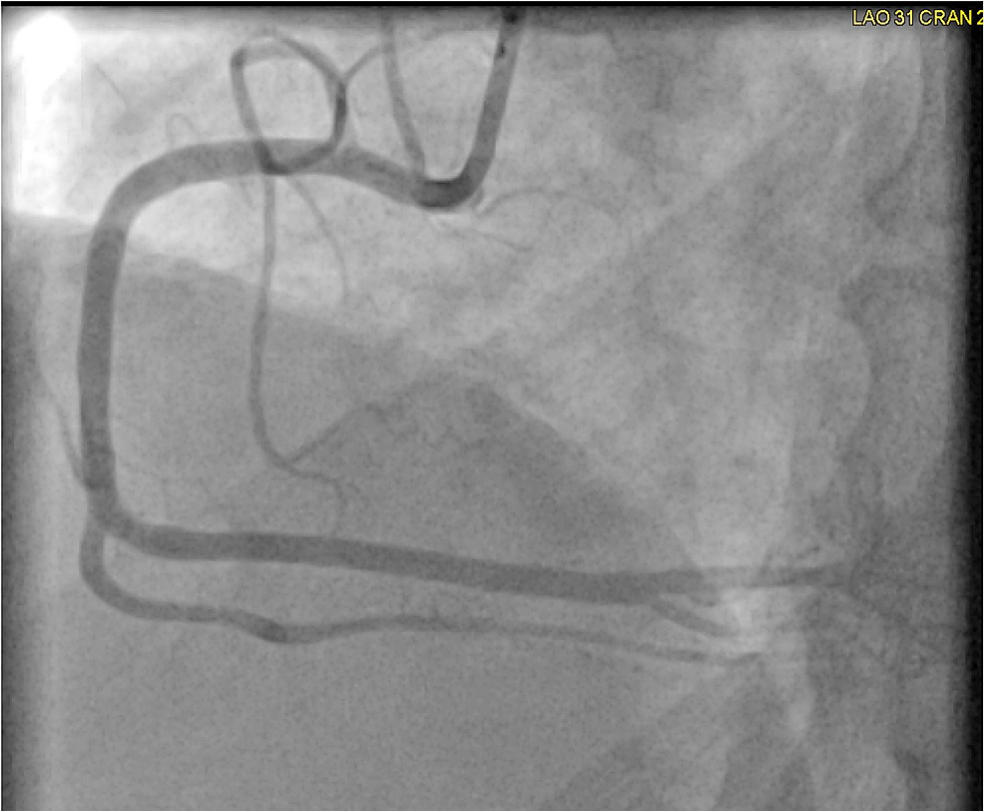
Coronary angiogram demonstrating no right coronary artery obstruction after nitroglycerin administration

Further history revealed the patient was in town to attend her brother’s funeral, and she had had no prior history of myocardial infarction or stroke. The patient denied tobacco, alcohol, and recreational drug use, and she was only taking methotrexate for her rheumatoid arthritis. Due to the patient’s anginal symptoms and ECG changes concerning for ischemia and elevated troponin, cardiac catheterization was performed. Left coronary artery circulation on her coronary angiogram did not demonstrate any significant obstruction (Figure 2). Her coronary angiogram showed a right coronary artery obstruction (Figure 3). The right coronary artery obstruction was resolved with nitroglycerin during the coronary angiogram (Figure 4). The patient was then diagnosed with Prinzmetal’s angina based on the visualization of coronary spasm and its resolution after nitroglycerin on the coronary angiogram (Figure 4). The patient was started on low-dose amlodipine, isosorbide mononitrate, aspirin, statin, and as-needed nitroglycerin for the Prinzmetal’s angina. She was later discharged in stable condition with the above treatment regimen and recommendation of avoiding smoking, beta-blockers, and mental stress to prevent future recurrences of her angina.

## Discussion

Coronary arteries can usually balance the blood demand and supply to the cardiac tissues by either vasodilation or vasoconstriction. In vasospastic angina, abnormal vasoconstriction causes sudden and transient occlusion of one or more coronary arteries, which triggers observed symptoms in patients with VSA [4-6]. The exact pathogenesis of coronary artery spasm is not fully understood; several different mechanisms have been suggested, including vascular smooth muscle cell hyperreactivity, altered autonomic nervous system response, endothelial dysfunction, oxidative stress, low-grade inflammation, or magnesium deficiency. Vascular smooth muscle hyperreactivity is believed to be the main contributor of coronary spasm, and it is caused by an increase in Rho-kinase activity, which enhances Ca2+ mediated myosin light chain phosphorylation. This myosin light chain phosphorylation promotes vasoconstriction and coronary spasm. The imbalance between the sympathetic and parasympathetic nervous systems may play an important role in coronary artery spasms. Acetylcholine released by the parasympathetic nervous system can induce smooth muscle spasms, which helps explain the increased frequency of VSA at rest or midnight when vagal tone is at its highest levels. Endothelium-produced nitric oxide promotes vasodilatation by suppressing the release of vasoconstricting agents, including endothelin-I and angiotensin II. Therefore, the dysfunction of the endothelium can cause vasoconstriction due to nitric oxide deficiency. Oxidative stress is another prominent trigger for VSA, as oxygen free radicals can degrade nitric oxide and damage smooth muscle endothelial cells causing coronary artery vessel spasm. Additionally, inflammatory markers, such as C-reactive protein, intercellular adhesion molecule-1, interleukin-6, and monocyte chemoattractant protein-1, are thought to contribute to VSA, as these biomarkers are elevated in patients with vasospastic angina [4-5]. This correlation was supported by the onset of coronary artery spasm when interleukin-B was administered [5]. In addition, smoking is a major risk factor of VSA, as cigarettes can cause a chronic inflammatory state in smokers.

Normal ECG may appear at the beginning of coronary artery spasm or mild spasm. However, the sudden vasoconstriction and transient occlusion of the coronary artery can cause ECG changes in patients with VSA. The most common ECG changes are symmetrical peaked T waves in 50% of patients. Other ECG changes include ST-segment elevation or depression, increased height and width of the R wave, negative T wave, decreased magnitude or disappearance of S wave, or U wave appearance [1,4]. Arrhythmias can also be seen during VSA such as supraventricular tachyarrhythmias, atrioventricular block, ventricular premature contractions, ventricular tachycardia or fibrillation, and asystole.

Vasospastic angina can be diagnosed using three considerations according to the Coronary Vasomotion Disorders International Study Group, including (1) angina responding to nitrate or calcium channel blockers, (2) transient ECG changes during spontaneous angina episodes, and (3) provocative or spontaneous coronary artery spasm, which induce artery occlusion associated with angina episode and ischemic ECG changes (Table 1). Lifestyle changes and pharmacological treatment are two mainstay recommendations for VSA management. With endothelial dysfunction and oxidative stress effects on the endothelial function being two of the main etiologies for VSA, smoking cessation is important in VSA patients. Avoiding risk factors of mental stress, alcohol consumption, cocaine use, beta-blockers, or magnesium deficiency can also help minimize exacerbations. Several pharmacological agents are also utilized to treat VSA such as calcium channel blockers, nitrate, Rho-kinase inhibitors, and others. Calcium channel blockers are used at first-line treatment for VSA, with several studies showing their effectiveness in decreasing angina frequency [4,6-7]. Several randomized trials have demonstrated the efficiency of nitrates in reducing VSA symptoms; thus, nitrates are recommended as a second option after calcium channel blockers. An increase in Rho-kinase activity is thought to be one of the mechanisms for vasospasm, so Rho-kinase inhibitors, such as Fasudil, can be used to treat VSA. Several other pharmacological agents, such as statins, nicorandil, or alpha 1-adrenergic receptor antagonists, are being studied as possible treatments for coronary artery spasms.

**Table 1:** Criteria for diagnosing vasospastic angina

1. Nitrate-responsive angina (during a spontaneous episode, with at least one of the following)
a. Rest angina, especially between night and early morning
b. Marked diurnal variation in exercise tolerance, reduced in morning
c. Hyperventilation can precipitate an episode
d. Calcium channel blockers (but not beta-blockers) suppress episodes

(2) Transient ischemic ECG changes (during a spontaneous episode, including any of the following in at least two contiguous leads)
a. ST-segment elevation ≥0.1 mV
b. ST-segment depression ≥0.1 mV
c. New negative U waves

(3) Coronary artery spasm
a. Defined as transient total or subtotal coronary artery occlusion (>90% constriction) with angina
b. Ischemic ECG changes either spontaneously or in response to a provocative stimulus (typically acetylcholine, ergot, or hyperventilation)

Our patient was first evaluated for possible STEMI due to her symptoms of chest pain, chest heaviness, diaphoresis, and syncope along with moderately elevated troponin I levels and elevated ST-segment on ECG. However, STEMI was ruled out, and the diagnosis of vasospastic angina was eventually made after normal cardiac catheterization. The patient’s VSA was possibly triggered or exacerbated by the emotional stress that she had endured after her brother's passing. She responded well to the therapy of amlodipine, isosorbide mononitrate, aspirin, statin, and nitroglycerin. Our favorable patient response to the pharmacological regimen helps support the benefits of the agents discussed above in managing VSA patients.

## Conclusions

Recognizing, diagnosing, and treating chest pain accurately is very important among clinicians, as it is one of the most commonly presenting symptoms among patients. It is especially crucial in patients with clinical symptoms and laboratory findings suggestive of ACS, as these cardiovascular etiologies can post serious complications or even death to the patients. Therefore, understanding the clinical presentation, diagnosis, and management of vasospastic angina is equally essential, as it will aid us in properly managing patients with chest pain.
